# Effects of Acute Hypoxia on Lactate Thresholds and High-Intensity Endurance Performance—A Pilot Study

**DOI:** 10.3390/ijerph18147573

**Published:** 2021-07-16

**Authors:** Martin Faulhaber, Katharina Gröbner, Linda Rausch, Hannes Gatterer, Verena Menz

**Affiliations:** 1 Department of Sport Science, University Innsbruck, 6020 Innsbruck, Austria; K.Groebner@student.uibk.ac.at (K.G.); Linda.Rausch@uibk.ac.at (L.R.); Verena.Menz@uibk.ac.at (V.M.); 2 Austrian Society of Alpine and High-Altitude Medicine, 6414 Mieming, Austria; 3 Institute of Mountain Emergency Medicine, Eurac Research, 3910 Bolzano, Italy; Hannes.Gatterer@eurac.edu

**Keywords:** anaerobic threshold, high altitude, maximal lactate steady state

## Abstract

The present project compared acute hypoxia-induced changes in lactate thresholds (methods according to Mader, Dickhuth and Cheng) with changes in high-intensity endurance performance. Six healthy and well-trained volunteers conducted graded cycle ergometer tests in normoxia and in acute normobaric hypoxia (simulated altitude 3000 m) to determine power output at three lactate thresholds (P_Mader_, P_Dickhuth_, P_Cheng_). Subsequently, participants performed two maximal 30-min cycling time trials in normoxia (test 1 for habituation) and one in normobaric hypoxia to determine mean power output (P_mean_). P_Mader_, P_Dickhuth_ and P_Cheng_ decreased significantly from normoxia to hypoxia by 18.9 ± 9.6%, 18.4 ± 7.3%, and 11.5 ± 6.0%, whereas P_mean_ decreased by only 8.3 ± 1.6%. Correlation analyses revealed strong and significant correlations between P_mean_ and P_Mader_ (r = 0.935), P_Dickhuth_ (r = 0.931) and P_Cheng_ (r = 0.977) in normoxia and partly weaker significant correlations between P_mean_ and P_Mader_ (r = 0.941), P_Dickhuth_ (r = 0.869) and P_Cheng_ (r = 0.887) in hypoxia. P_Mader_ and P_Cheng_ did not significantly differ from P_mean_ (*p* = 0.867 and *p* = 0.784) in normoxia, whereas this was only the case for P_Cheng_ (*p* = 0.284) in hypoxia. Although investigated in a small and select sample, the results suggest a cautious application of lactate thresholds for exercise intensity prescription in hypoxia.

## 1. Introduction

Endurance training sessions under hypoxic conditions are part of altitude training concepts for competitive athletes [1], as well as in preventive and therapeutic settings [2,3]. The positive effects of hypoxia application on sport performance and health outcomes have been extensively described in literature, but there are also negative reports [4,5] that should not be ignored, especially since negative health effects (e.g., an increased mechanic stress against the cerebral vessel wall) are also suspected [5]. The determination of exercise intensity zones plays a key role in regulating training adaptations and preventing under or over strain. Anaerobic threshold concepts are very popular to prescribe intensity zones for endurance training; however, it remains unclear whether such concepts are still valid under hypoxic conditions. Despite its practical relevance, scientific literature dealing with anaerobic threshold concepts in hypoxia is scarce and, for example, ventilatory thresholds seem to be more reduced compared to lactate thresholds [6]. Thus, it remains unclear if intensity zones based on lactate or ventilatory threshold concepts are adequate tools for training prescription in hypoxia.

Recently, Weckbach et al. [7] reported that peak power output (P_max_) and power output at different lactate thresholds (LT), derived from incremental exercise testing, were significantly reduced in acute hypoxia (2650 m) compared to low altitude. Interestingly, the presented data revealed that the reduction in LT power output was more pronounced compared to P_max_ by 20 to 90% depending on the LT concept [7]. However, it remains unclear whether the reduction in LT also reflects a similar decrement in high-intensity endurance performance. If hypoxia-induced changes in LT differ markedly from changes in maximal steady-state performance, the validity of these threshold concepts for exercise prescription under acute hypoxic conditions would be limited. The present pilot study should contribute to filling this knowledge gap and, therefore, compared different LTs, derived from incremental exercise testing, with high-intensity endurance performance in normoxic and acute hypoxic conditions. Based on a previous study evaluating endurance performance at 3200 m [8] and data of Weckbach et al. at 2650 m [7], we hypothesised that power output at the lactate thresholds would be more impaired by an acute exposure to hypoxia compared to endurance performance.

## 2. Materials and Methods

### 2.1. Participants and Study Design

Six (three females, three males) students (age: 25 ± 3 years; body height: 176 ± 3 cm; body weight: 71 ± 5 kg; altitude of residence: 677 ± 134 m) from the Department of Sport Science (University of Innsbruck) volunteered to participate in this pilot study. All of them were regularly active for more than 3 h per week including various disciplines (as is common for active sport students), but none of them were competitive endurance athletes. Participants completed a routine health screening using an adapted physical activity readiness questionnaire (PAR-Q) before inclusion in the study. Medical clarification by a physician was undertaken if the PAR-Q identified specific issues that required further investigation. Exclusion criteria were pre-existing acute or chronic diseases, pregnancy or lactation and regular smoking of more than five cigarettes per day.

Participants were informed about the experimental details and gave informed consent before commencing the study. The study was carried out in conformity with the ethical standards laid down in the 1975 Declaration of Helsinki. Since the study was designed as a pilot project the identical protocol of the subsequent main study was approved by the Board for Ethical Questions in Science of the University of Innsbruck, Austria (Certificate of good standing, 12/2021).

**Figure 1:**
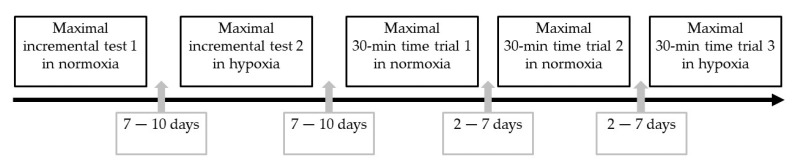
Experimental design including the sequence of the tests and the breaks between tests.

The study was designed as a within-subject design (without cross-over). Participants conducted five cycle ergometer (Cyclus 2, RBM, Leipzig, Germany) tests in a fixed order (Figure 1). Tests 1 and 2 were maximal incremental tests in normoxic (test 1) and hypoxic conditions (test 2). Test 1 and 2 were separated by a recovery period of 7 to 10 days. Tests 3 to 5 were maximal 30-min time trials in normoxic (tests 3 and 4) and hypoxic conditions (test 5), and tests were separated by recovery periods of 2 to 7 days. Test 3 served for habituation and was not included into statistical analyses. Adjustments of the cycle ergometer (e.g., saddle height) were fixed before the first test and kept constant for the subsequent tests. All tests took place in the laboratories of the University of Innsbruck (Department of Sport Science, 590 m). Tests under hypoxic conditions were conducted in a normobaric hypoxic chamber (LowOxygen Systems, Berlin, Germany) adjusted at an inspiratory fraction of oxygen of 15.4% corresponding to a simulated altitude of about 3000 m. The hypoxic system provides a high air flow keeping the inspiratory fraction of oxygen constant and to avoid an excessive increase in inspiratory fraction of carbon dioxide as reported in previous studies [9,10,11].

### 2.2. Tests and Measurements

#### 2.2.1. Maximal Incremental Tests

Participants rested for about 5 min in a sitting position on the cycle ergometer before resting parameters were taken. Workload started at 80 W for female and 100 W for male participants and was increased by 20 W every 3 min until subjective exhaustion. Heart rate (M430, Polar, Vienna, Austria) was monitored. Blood lactate concentrations (Super GL Ambulance, Dr. Müller Gerätebau, Freital, Germany) were analysed from capillary blood samples taken from the hyperaemised earlobe at the end of the resting phase, during the last 30 s of each stage and about 3 min after cessation of the test. P_max_ was defined as the last completed work rate plus the fraction of time spent in the final uncompleted work rate multiplied by 20 W [12]. Maximal heart rate (HR_max_) was defined as the highest 5-s average, and maximal blood lactate concentration (BLA_max_) was considered as the value of the last sample about 3 min after test termination. Heart rate and blood lactate values were transferred to an automated software (winlactat, Mesics, Münster, Germany) to determine LTs. In accordance with the study of Weckbach et al. [7], we selected three different methods for the detection of the LT: (a) fixed 4-mmol/L blood lactate concentration according to Mader et al. [13], (b) lactate concentration of 1.5 mmol/L above the minimal lactate equivalent according to Dickhut et al. [14], and (c) maximal perpendicular distance from the blood lactate concentration curve to the line drawn from start- to endpoint (also known as D_max_ method) according to Cheng et al. [15]. Outcome parameters of the maximal incremental tests were P_max_, HR_max_ as well as power output and heart rate at the three LT (P_Mader_, P_Dickhuth_, P_Cheng_ and HR_Mader_, HR_Dickhuth_, HR_Cheng_).

#### 2.2.2. Maximal 30-min Time Trials

Time trials were conducted as described in detail in previous studies [8,16]. In brief: testing began with warm-up periods of 5 min at 100/150 W (females/males) followed by 5 min at 150/200 W (females/males). Then, the cycle ergometer was shifted to a fixed pedal force so that pedalling at 100 rpm produced about 70% of P_max_ (determined by the maximal incremental test in normoxia). Participants were encouraged to choose a maximal pedalling rate that could be maintained for the respective test duration. Heart rate was measured continuously (chest belt, Polar, Austria), and capillary blood samples were taken from the hyperaemised earlobe after 7 and 27 min to determine blood lactate concentrations (BLA_7_ and BLA_27_) (Super GL Ambulance, Dr. Müller Gerätebau, Germany). Outcome parameters were mean power output (P_mean_) and mean heart rate (HR_mean_), which were automatically calculated by the software of the ergometer, BLA_7_ and BLA_27_.

### 2.3. Statistics

Statistical analyses were performed using SPSS 24.0 (IBM, Vienna, Austria). Data were checked for normal distribution using the Shapiro–Wilk test. Since data were normally distributed (except for BLA_7_ and BLA_27_), paired t-tests (Wilcoxon rank tests for BLA_7_ and BLA_27_) were used to compare outcome parameters in normoxic versus hypoxic conditions. In the next step, focusing on power output parameters separated for normoxic and hypoxic conditions, Pearson correlation analyses between power output at the LT and P_mean_ were performed, and potential differences were tested by paired t-tests. In addition, the hypoxia-related reductions in power output at the LTs and in P_mean_ were compared with paired t-tests to test whether lactate thresholds are affected differently by acute hypoxia compared to high-intensity endurance performance. The level of significance was set at *p* < 0.05. Data are presented as means ± SD.

## 3. Results

The results of the maximal incremental tests revealed a significant decrease in P_max_ by approximately 12% from normoxia to hypoxia (249 ± 25 versus 221 ± 36 W, *p* = 0.005). HR_max_ (189 ± 10 versus 186 ± 8 bpm, *p* = 0.217) and BLA_max_ (11.9 ± 2.5 versus 13.1 ± 0.9, *p* = 0.166) did not significantly differ from normoxia to hypoxia.

Power output and heart rate at the different LT and parameters of the maximal 30-min time trials are shown in Table 1. Power output decreased for all threshold concepts by about 12 to 19%, whereas heart rate values showed small (0 to 5%) and not significant changes from normoxia to hypoxia. P_mean_ decreased significantly from normoxia to hypoxia by approximately 8%, and HR_mean_ was slightly lower in hypoxia compared to normoxia, although not statistically significant. Blood lactate concentrations during the time trials did not significantly differ between normoxia versus hypoxia (BLA_7_: 6.1 ± 2.2 versus 6.3 ± 2.2 mmol/L, *p* = 0.873; BLA_27_: 8.9 ± 2.5 versus 9.4 ± 2.0 mmol/L, *p* = 0.385).

**Figure 2:**
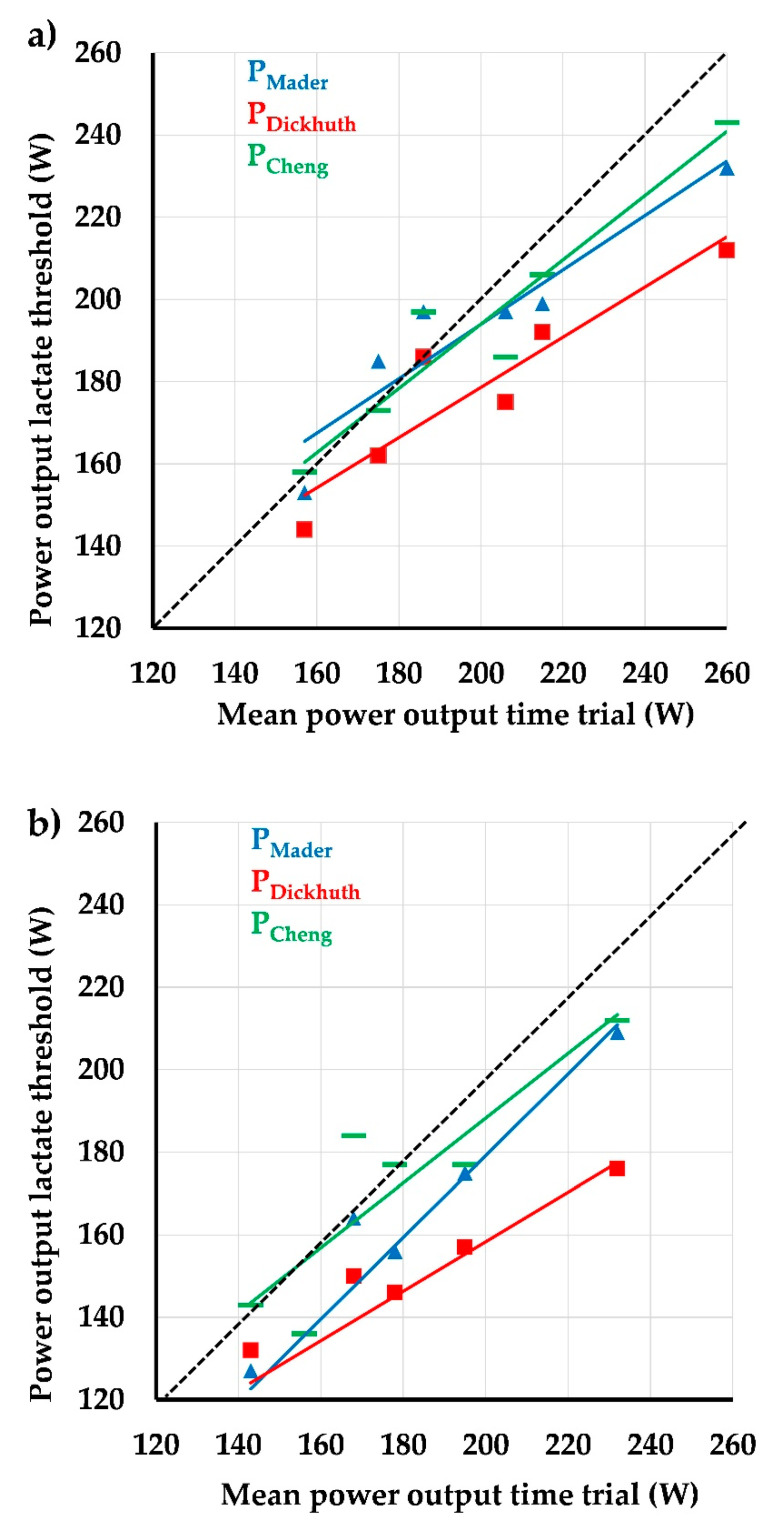
Association between mean power output during the time trial and power output at three different lactate thresholds (blue = P_Mader_, red = P_Dickhuth_, green P_Cheng_) [11,12,13]. Data are presented for normoxic (upper panel) und hypoxic conditions (lower panel). (**a**): normoxic conditions; (**b**): hypoxic conditions.

Correlation analyses for normoxia (Figure 2a) data revealed strong and significant correlations for power output at the three LT and P_mean_ (P_Mader_: r = 0.935, *p* = 0.006; P_Dickhuth_: r = 0.931, *p* = 0.007; P_Cheng_: r = 0.977, *p* = 0.001). Furthermore, P_Mader_ and P_Cheng_ did not significantly differ from P_mean_ (*p* = 0.867 and *p* = 0.784 respectively), whereas P_Dickhuth_ was significantly lower compared to P_mean_ (*p* = 0.045). With respect to hypoxic conditions (Figure 2b), we also found significant correlations, but for P_Dickhuth_ and P_Cheng,_ we found slightly weaker correlations (P_Mader_: r = 0.941, *p* = 0.005; P_Dickhuth_: r = 0.869, *p* = 0.024; P_Cheng_: r = 0.887, *p* = 0.019). P_Mader_ and P_Dickhuth_ were significantly lower compared to P_mean_ (*p* = 0.007 and *p* = 0.005), whereas there was no significant difference for P_Cheng_ (*p* = 0.284).

**Figure 3:**
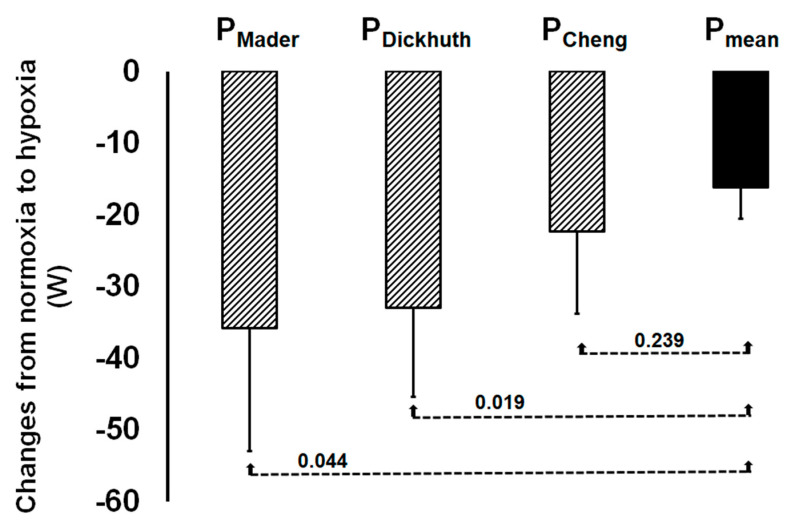
Hypoxia-related changes in power output at the three lactate thresholds (P_Mader_, P_Dickhuth_, P_Cheng_) and in mean power output during the maximal 30-min time trial (P_mean_) [13,14,15]. *p*-Values refer to a comparison to P_mean_. Values are means ± SD.

Comparing hypoxia-related impairments in power output at the three LT and in P_mean,_ significant differences were found for P_Mader_ and P_Dickhuth_ but not for P_Cheng_ (Figure 3).

## 4. Discussion

The presented data show that acute hypoxia impaired power output at different LT and P_mean_ as expected. In accordance with our hypothesis, the results also demonstrate that the reduction in P_mean_ was clearly lower (approximately 8%) compared to lactate thresholds estimations (12% to 19%). Correlations between power output at the LT and P_mean_ were found in normoxic as well as in acute hypoxic conditions. However, hypoxia-related decreases in power output at two LT (P_Mader_ and P_Dickhuth_) were significantly greater compared to the hypoxia-related decrease in P_mean_ resulting in significant differences to P_mean_ in hypoxia.

Although LT are often validated for specific exercise protocols, exercise modes and populations, the observed strong correlations in normoxic conditions between power output at the LT (P_Mader_, P_Dickhuth_, P_Cheng_) and P_mean_ were well documented [17] and in accordance with our findings. The method of Dickhuth et al. [14] was designed to detect the first rise in blood lactate concentration and can be categorised as an aerobic lactate threshold [17]. This observation was also reported in the review article of Faude et al. [17], and, therefore, the significant underestimation of P_mean_ in our study was not surprising. However, this underestimation of P_mean_ does not limit the application of this LT in exercise prescription by model-specific intensity zones.

Regarding the hypoxia-induced changes in power output, the three LT showed more pronounced reductions compared to P_mean_. From a practical point of view, the application of LT-based intensity zones, although fitting in normoxic conditions, can result in an underestimation of endurance capacity and therefore sub-optimal or even ineffective training loads when determined and applied in acute hypoxic conditions. Since the method according to Cheng et al. [15] only slightly underestimated P_mean_ and, furthermore, showed a strong correlation to P_mean_ under hypoxic conditions, it seems that exercise intensity prescriptions based on this method may be more robust against hypoxic-related underestimations. Based on the observation that resting lactate concentrations are not influenced by moderate hypoxia (e.g., 3000 m) but are markedly pronounced during exercise at the same absolute intensity level [18,19], the following explanations may be reasonable: LT models, defined by a fixed blood lactate concentration (i.e., 4 mmol/L) or adding a fixed value (i.e., 1.5 mmol/L) to an individual minimum may be directly affected by changes in the absolute lactate values (e.g., under acute hypoxia). In contrast, the LT model by Cheng et al. [15] also considers the shape of the blood lactate curve and thus the individual lactate kinetics, which seems to be a crucial point in evaluating exercise capacity [20].

To the best of our best knowledge, this is the first study comparing hypoxia-related changes in lactate thresholds with changes in endurance performance (i.e., 30-min time-trial performance). The inclusion of a first time trial for familiarisation improved the validity of the hypoxia-related changes in the second and third time trial because it was shown that time-trial performance improves from a first to a second test [16]. The small sample size and the resulting susceptibility for individual outliers represent the main limitation of the present study. For example, the correlation analysis in hypoxia revealed r = 0.887 for P_Cheng_ versus P_mean_ (*p* = 0.019). However, the exclusion of one person (a statistical borderline outlier) would result in r = 0.959, (*p* = 0.010, *n* = 5). In addition, this experiment was conducted in a specific group of healthy subjects with an above-average fitness level and results cannot be directly transferred either to elite athletes or to specific patient groups.

## 5. Conclusions

According to the hypothesis, power output at the lactate thresholds were more impaired by an acute exposure to hypoxia compared to high-intensity endurance performance reaching statistical significance for the methods of Mader et al. and Dickhuth et al. In conclusion, the application of LT for exercise intensity prescription in hypoxia, even when determined under such conditions, may be prone to errors. The results of this pilot study should provide a basis for future larger-scale investigations dealing with this topic in different target groups.

## Figures and Tables

**Table 1:** Power output and heart rate at different lactate thresholds and during maximal 30-min time trials in normoxia and hypoxia. Values are means ± SD. *p*-Values refer to a comparison of normoxia and hypoxia.

	Normoxia	Hypoxia	Difference (%)	*p*-Value
Lactate thresholds based on stepwise maximal cycle ergometries				
P_Mader_ (W)	194 ± 26	158 ± 33	−18.9 ± 9.6	0.004
HR_Mader_ (bpm)	167 ± 8	160 ± 12	−4.5 ± 6.8	0.154
P_Dickhuth_ (W)	179 ± 24	146 ± 22	−18.4 ± 7.3	0.001
HR_Dickhuth_ (bpm)	161 ± 8	153 ± 16	−5.1 ± 7.0	0.140
P_Cheng_ (W)	194 ± 30	172 ± 28	−11.5 ± 6.0	0.005
HR_Cheng_ (bpm)	167 ± 9	167 ± 11	+0.3 ± 8.0	0.977
Maximal 30-min time trials				
P_mean_ (W)	195 ± 34	179 ± 32	−8.3 ± 1.6	<0.001
HR_mean_ (bpm)	175 ± 12	170 ± 8	−2.7 ± 4.4	0.203

P, power output; HR, hear rate. Lactate thresholds were determined according to the methods of Mader et al. (_Mader_), Dickhuth et al. (_Dickhuth_) and Cheng et al. (_Cheng_) [13,14,15]. Pmean, mean power output; HRmean, mean heart rate.

## Data Availability

The data are not publicly available due to ethical considerations on preserving the anonymity of study participants.
